# The Role of Natural Language Processing during the COVID-19 Pandemic: Health Applications, Opportunities, and Challenges

**DOI:** 10.3390/healthcare10112270

**Published:** 2022-11-12

**Authors:** Mohammed Ali Al-Garadi, Yuan-Chi Yang, Abeed Sarker

**Affiliations:** 1 Department of Biomedical Informatics, Vanderbilt University Medical Center, Nashville, TN 37240, USA; 2 Department of Biomedical Informatics, School of Medicine, Emory University, Atlanta, GA 30322, USA

**Keywords:** COVID-19, natural language processing, health applications, machine learning, deep learning

## Abstract

The COVID-19 pandemic is the most devastating public health crisis in at least a century and has affected the lives of billions of people worldwide in unprecedented ways. Compared to pandemics of this scale in the past, societies are now equipped with advanced technologies that can mitigate the impacts of pandemics if utilized appropriately. However, opportunities are currently not fully utilized, particularly at the intersection of data science and health. Health-related big data and technological advances have the potential to significantly aid the fight against such pandemics, including the current pandemic’s ongoing and long-term impacts. Specifically, the field of natural language processing (NLP) has enormous potential at a time when vast amounts of text-based data are continuously generated from a multitude of sources, such as health/hospital systems, published medical literature, and social media. Effectively mitigating the impacts of the pandemic requires tackling challenges associated with the application and deployment of NLP systems. In this paper, we review the applications of NLP to address diverse aspects of the COVID-19 pandemic. We outline key NLP-related advances on a chosen set of topics reported in the literature and discuss the opportunities and challenges associated with applying NLP during the current pandemic and future ones. These opportunities and challenges can guide future research aimed at improving the current health and social response systems and pandemic preparedness.

## 1. Introduction

**Figure 1:**
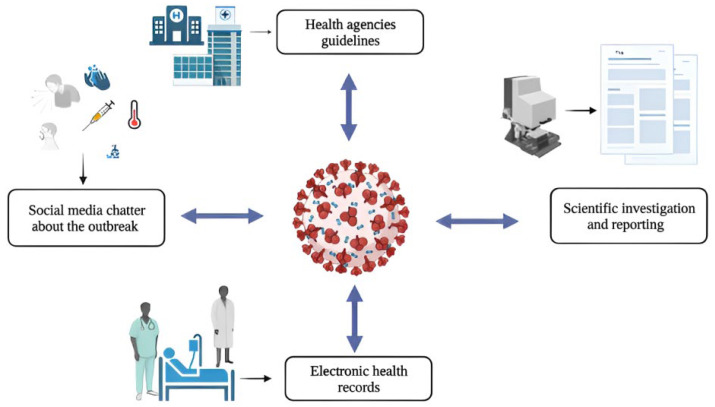
Important text-based information generated from distinct sources during the COVID-19 pandemic.

**Figure 2:**
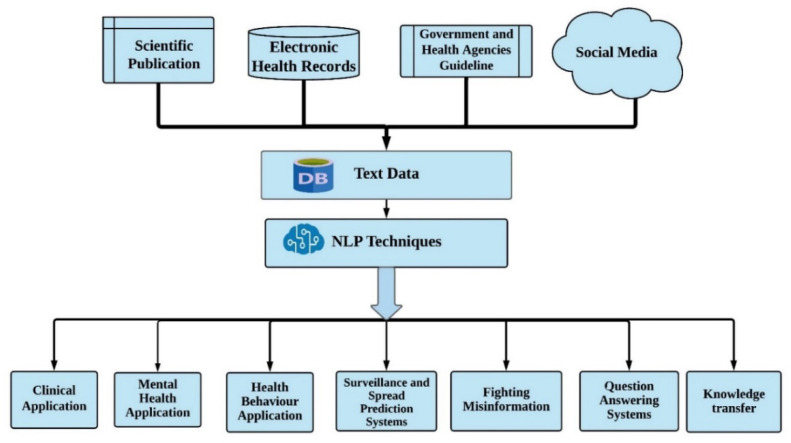
Summary of applications of NLP methods for COVID-19 reviewed in this paper. Distinct information sources are shown at the top.

During a global health crisis such as the current COVID-19 pandemic, healthcare systems need practical solutions that can help provide effective care services and mitigate its impact on society. Outbreaks of novel diseases exert considerable pressure on public health and hospital systems [1,2]. Unlike past pandemics, however, the current one has occurred at a time when healthcare systems and public health agencies have access to large-scale data. Thus, the challenges posed by the crisis offer an opportunity to improve public health systems through the use of innovative technologies such as data-driven artificial intelligence (AI) [3]. One subset of AI technologies with enormous potential is natural language processing (NLP), particularly due to the large volumes of free-text data that are currently available and continuously generated through different channels, such as electronic health records (EHRs), published medical literature, and social media. The NLP of EHRs, for example, can help medical practitioners identify patterns in free-text clinical big data generated by COVID-19 patients, and/or discover the latent factors influencing their long-term outcomes [4]. The NLP of social media data may help address challenges associated with the COVID-19 *infodemic*, which refers to the massive spread of health disinformation and misinformation during the pandemic [5]. NLP applied to social media data related to COVID-19 may also help monitor people’s mental health during the evolution of the pandemic, act as disease surveillance systems, and help to understand the psychological and sociological processes that can influence people to follow suggested health behaviors for the COVID-19 pandemic. NLP may be applied to the scientific literature, which is evolving fast during the pandemic, to establish real-time evidence-based question-and-answer systems that can automatically translate the latest scientific knowledge to several languages to disseminate the findings globally [6]. It can also help frontline physicians address problems associated with information overload [7,8]. Figure 1 and Figure 2 provide visual summaries of the data sources and opportunities discussed in the following sections of this paper.

A number of recent reviews have addressed topics broadly at the intersection of AI and COVID-19, and some have focused specifically on NLP. The literature review by Grabar and Gruin [9] focused on NLP advances in 2020 and specifically discussed three papers in detail, which they considered the best papers in their review. Several reviews have included NLP as a topic within the broader sphere of AI [10,11,12,13]. A recent scoping review discussed the potential use of AI methods, including NLP approaches, during the COVID-19 pandemic [14]. Another scoping review emphasized the critical role of social media in circulating health information and dealing with pandemic-related infodemics and misinformation [15]. Similarly, a prior systemic review discussed different social media uses for public health [16]. Other similar previous review articles discussed deep learning applications for COVID-19 in general [17,18,19,20,21], or NLP use for COVID-19 in particular [22]. Most of the reviewed deep learning approaches for COVID-19 are focused on image classification applications [17,18,19,20,21]. The review article in [22] discussed several pre-trained NLP models with use cases for a sentiment analysis associated with COVID-19 vaccination. However, the review we present in this paper, unlike the abovementioned recent reviews, thoroughly appraises a carefully selected set of recent papers on the application of NLP approaches during the COVID-19 pandemic, in order to improve pandemic preparedness and response. We include studies involving diverse text data sources such as EHRs, official agency guidelines, social media, and scientific publications, and cover many relevant applications (see Figure 1 and Figure 2). However, our review is not designed to be a systematic review. Instead, the focus is to select a small set of important papers on several chosen topics and discuss their contributions, limitations, and potential extensions. On top of highlighting these papers, we provide future research directions. Our review follows the structure of several previous review articles [23,24,25,26,27,28]. The rest of the paper is written as follows: in Section 2, we discuss NLP methods applied to clinical notes encoded in EHRs. Section 3 covers studies that focused on the application of NLP approaches to understand individuals’ mental health during the pandemic. Section 4 discusses the studies that have proposed NLP approaches to study people’s health behaviors during the pandemic. Section 5 deliberates the studies that investigated the potential utilization of NLP techniques on social media data to build COVID-19 surveillance and outbreak prediction systems. Section 6 reviews studies that examine NLP approaches to tackle the problem of misinformation during the COVID-19 pandemic. Section 7 discusses studies that utilized NLP approaches to construct real-time question-answering (QA) systems based on the scientific literature, which can effectively disseminate information during an urgent situation such as the COVID-19 pandemic. Section 8 summarizes studies that employed NLP methods to translate scientific findings from different languages. Section 9 builds on the previous ones and presents the issues, challenges, and future directions of the NLP applications during a pandemic. We summarize the motivation, significance, and contributions of this review in the following subsections.

### 1.1. Motivation

Innovations in NLP approaches offer an opportunity to improve current healthcare and public health systems. Large volumes of free-text data are available and continuously generated through various channels, such as EHRs, published medical literature, and social media. The primary motivation of this review is to discuss some of the many uses of NLP-based technologies that can enhance pandemic preparedness and response (such as for COVID-19) and their potential applications in unpredictable future pandemics.

### 1.2. Significance

This review discusses a set of crucial uses of NLP approaches that can improve pandemic preparedness and response and which may be useful in unforeseen future pandemics. Lessons learned from the current use of NLP applications during the COVID-19 pandemic are also presented. Furthermore, we discuss several opportunities and remaining challenges associated with the application of NLP during a pandemic. These opportunities and challenges can be used as a guide in identifying future research directions and advancing the existing health and social response structures and pandemic readiness systems.

### 1.3. Contributions

The key contributions of this survey are as follows:

- A review of the various applications of NLP that can improve pandemic preparedness and response, and their potential use in future pandemics.
- A deliberation of lessons learned in different NLP application areas in each section, followed by comparisons and a summary of reviewed studies.
- A detailed presentation of research challenges and potential future directions. The challenges we present can be used as a guide for future studies that seek to advance the present health and social response systems and pandemic preparedness.

## 2. NLP for Electronic Health Records (EHRs)

The comprehensive adoption of EHRs in healthcare produces large real-world data that introduce new opportunities for critical clinical research. EHRs contain structured and unstructured data; the latter are typically referred to as clinical notes. As a significant volume of valuable clinical information is available in clinical notes, NLP techniques can be used for the real-time extraction of information from clinical free text. The utilization of EHRs for healthcare or scientific research requires data to be encoded and comparable [29]. In general, the role of NLP for this type of data is to convert unstructured data (i.e., free text data) into structured information that can be readily accessed and used. The key advantage of NLP applications for such data is that they enable the prompt utilization of extensive clinical data [2], allowing the use of EHRs for patients with novel diseases as soon as they are included in the system [30]. Although NLP application has been frequently recommended [31], such claims have not been tested in real time [30]. Thus, the present COVID-19 pandemic, with all of its challenges, can provide an opportunity to develop and implement real-time NLP models for EHRs with significant practical applications. The usefulness and applicability of NLP to clinical text [30] in response to emergencies have been evaluated with the main question of whether applying NLP models to unstructured textual information can yield clinically actionable knowledge. The outcomes indicate that NLP models can be developed rapidly to serve a novel disease domain and extract valuable information [30]. When combined with structured data, the extracted knowledge is often able to increase the sample size satisfactorily to observe treatment effects that may not have been previously statistically detectable.

NLP models may serve as the main components of clinical AI systems that extract self-reported symptoms from individuals’ audio or video recordings of clinic visits. A recording generally presents more informative facts about patient-reported symptoms compared to other sources. Recordings of clinic visits prepared at scale and combined with data from EHRs can enhance NLP models, thereby quickly creating patient-level clinical phenotypes of COVID-19 [32]. If clinical consultations are recorded and NLP models are effectively developed, benchtop virological findings can be better informed [32]. The potential role of NLP models to detect stroke during the COVID-19 pandemic from radiology reports has also been investigated [33]. The results demonstrated the potential of NLP approaches to automatically track acute or sub-acute ischemic stroke numbers for epidemiological studies. NLP models have also been developed to extract risk factors related to severe or non-severe COVID-19 from unstructured free text [34], and they showed promising results and the potential for real-time clinical applications.

NLP approaches have also been shown to be useful for extracting signs or symptoms of COVID-19 from clinical free text [35]. Owing to the importance of such NLP tasks, datasets such as the COVID-19 Annotated Clinical Text (CACT) have been created [36]. CACT is a dataset with annotations for COVID-19 diagnoses, testing, and symptoms that are used for training NLP models to detect annotated COVID-19 entities. Such datasets and others have enabled the development of machine learning (ML)-oriented NLP models. For instance, using a combination of NLP and ML methods enables the prediction of potential ICU admissions from the EHRs of patients with COVID-19 [37]. Another study used hospital discharge summary notes to develop an NLP pipeline to categorize the discharge dispositions of such patients [38]. Within the Department of Veterans Affairs (VA), a study developed an NLP system to extract possible positive COVID-19 cases from clinical text [39]. Detecting positive cases from clinical notes can help reduce the number of patients that laboratory-based surveillance methods may miss, and therefore, are not counted in the overall number of cases. Since EHRs in the VA contain data from hospitals across the United States, such a model can be useful for surveillance at the national level.

From the aforementioned papers, it is evident that with recent advances the application of NLP techniques in clinical notes can reveal new insights into real-time self-reported symptoms extraction, predicting potential ICU admissions, and improving pandemic prediction. The valuable information from these real-world data can aid research, healthcare systems, and regulatory activities. However, the characteristics of clinical notes pose many challenges for the application of NLP techniques, such as varying data quality, the difficulty of accurately de-identifying notes to protect patients’ privacy, and difficulties associated with interoperability.

## 3. NLP for Mental Health

During the COVID-19 pandemic, most governments around the globe implemented strict domestic quarantine policies to control the spread of the disease. Infringement on personal freedom, financial hardship, misinformation, and uncertainties about the new virus are among the significant stressors that have been reported to increase emotional distress and risks of psychiatric illnesses associated with COVID-19 [40]. The pandemic is associated with elevated levels of psychological distress which, in many cases, meet the threshold for clinical relevance. Thus, relieving the severe effects of COVID-19 on mental health has become a worldwide public health priority [41].

NLP models can promptly monitor public sentiments and emotions on a large scale [42,43]. The use of NLP techniques to understand the mental states of individuals through the analysis of their posts on social media platforms is increasing. This analysis of public commentaries, such as on Twitter, Reddit, and Facebook, can capture the users’ concerns, emotions, and mental states in real-time. A recent study applied NLP techniques to COVID-19-related data on Reddit to understand individuals’ mental health. The authors showed that NLP techniques have been helpful to reveal mental health complaints in real time, recognize vulnerable individuals, and detect rapidly rising mental health-related topics during COVID-19 [44]. The study shows that NLP techniques performed robustly in finding mental health complaints in real time, as well as identifying vulnerable groups and important mental health-related topics during the pandemic. As discovered by NLP techniques, several linguistic patterns of mental health status can serve as helpful indicators and clues for further investigation in clinical settings [44].

Another study that aims to provide a research resource for developing NLP models created the Emotion-Covid19-Tweet (EmoCT) dataset containing 1000 annotated English tweets used for NLP model training. In the dataset, English tweets are labeled as expressing *anger*, *anticipation*, *disgust*, *fear*, *joy*, *sadness*, *surprise*, and *trust* [45]. In a separate study, over 20 million COVID-19 tweets between January 28 and April 9, 2020 were used to examine the shift of public emotions during the early phases of the disease outbreak [46]. Fears about the unavailability of COVID-19 tests and medical supplies gradually turned into common discussion topics. *Sadness* was expressed in discussions about losing friends and family members, whereas topics related to *joy* were found to contain words of appreciation for good health [46]. In a similar direction, another study applied NLP techniques to explore 47 million COVID-19-related comments extracted from Twitter, Facebook, and YouTube. The findings showed that a total of 34 negative topics appeared, out of which 15 were related to COVID-19, specifically focusing on health, psychosocial, and social issues from the population health perspective. Furthermore, 20 positive topics were found, which were commonly related to public awareness, inspiration, gratitude, online learning, charity, spiritual support, innovative research, and a better environment [47].

NLP techniques can help to analyze real-time social media posts to understand temporal mental health dynamics associated with changes in COVID-19 regulations (such as national lockdowns). For instance, the correlation between temporal mental health dynamics and COVID-19 events was investigated in a study [19], and the results empirically demonstrated an association between the populations’ temporal mental health dynamics and national lockdowns. Such findings can be referenced as a second opinion during strategic decision making.

NLP approaches have also been applied to free-text notes from sources other than social media to assess mental health status. For example, research has analyzed the free text generated by college students through an application designed to help improve their mental health [48]. The study intended to understand the sentiments that students reveal on specific topics between pre- and post-COVID-19 periods. The findings disclosed that topics such as *Education* became remarkably less essential to students after the pandemic, whereas topics on *Health* became more imprinting and trending. Moreover, the students expressed more negative sentiments across all topics in post-COVID-19 discussions than before the pandemic [48].

The real-time monitoring of mental health during a pandemic is vital for public health agencies that strive to improve public awareness and reduce the negative impact of the pandemic on individuals’ mental health. From the literature, it is evident that NLP techniques can be used in near real-time mental health surveillance systems that can track, at a large scale, trends in people’s mental health statuses associated with news, guidelines, misinformation, and public health responses during distinct phases of the pandemic. However, the validity of observational social media research on mental health status is still a challenge, as discussed in previous research [49,50,51]. The challenges can introduce gaps that may limit the deployment of NLP techniques on social media data to predict mental health status in clinical and public health systems [51].

## 4. NLP for Understanding Health Behaviors

An important factor in the successful implementation of effective strategies to control the spread of an infectious disease is to understand the psychological and sociological processes that can influence people to follow recommended health behaviors. During the COVID-19 pandemic, one of the essential health behaviors was social distancing, which made it important to mine information on how seriously people followed the suggested government guidelines that were intended to reduce the spread of the virus [52]. Research has shown that NLP techniques and data from social media can construct useful models to understand health behaviors during the COVID-19 pandemic [52]. Large-scale social media text can be analyzed using NLP approaches to understand the discussions shared over social media and people’s reactions to specific guidelines. The conclusions and findings from such studies may help to rapidly inform public health policymakers and enable them to design corresponding strategies. The extracted information can also enable the analysis of public discourse on social distancing for use in future public health measures [53].

An analysis of Twitter data related to mask wearing revealed insights into social awareness of COVID-19 and its prevention [54]. For instance, past studies [54] suggested that high-profile users exert a significant influence (positive or negative) in spreading awareness about medical prevention approaches. Similarly, an NLP classifier [55] was developed to identify COVID-19 tweets that contained personal opinions about wearing masks. The study showed that the percentage of tweets related to anti-mask wearing was constant (approximately 10% of all tweets) during the study period (January to October 2020). The main justifications represented in anti-mask tweets were feeling physical discomfort, lack of effectiveness, and them being unnecessary or inappropriate for specific people or under certain circumstances. Anti-mask tweets were significantly less likely to cite scientific or official external information sources that supported their claims. Overall, combining social media data and NLP can help determine people’s perceptions of specific health issues (e.g., wearing masks) related to COVID-19 and provide public health policymakers with more insights to improve the interventions for the ongoing global pandemic.

NLP methods such as topic modeling and aspect-based sentiment analysis have been used to analyze topics in COVID-19-related tweets [56]. Trending topics on social media have been investigated versus the timing of implementation of interventions; they have been found to be highly correlated to public health behavior promotions such as physical distancing, handwashing, staying at home, and face covering. Applying NLP approaches to understand people’s behaviors or opinions about a public health event can be used in long-term plans to monitor public health campaigns that can help governments create effective communications. For instance, the literature has shown that developing an NLP model for understanding users’ opinions in social media towards vaccines (e.g., in favor, neutral, or against) can help to understand public concerns, and thus assist in designing effective communication that can help clarify their concerns and increase their awareness and trust [57].

One can conclude from the reviewed studies that the recent advances in NLP hold the potential to construct monitoring systems that can accurately and promptly track population-level health behavior associated with the guidelines and health agencies’ recommendations during a pandemic. However, the findings of several types of health behavior research that use NLP approaches applied to social media data are based on descriptive analyses. Such studies quantitatively show social data through numerical or graphical means. The descriptive analyses of tweets’ geographical distribution (for instance, examining if the recommended COVID-19 social distancing guideline is followed in a specific region) or descriptive analyses of the number of positive and negative opinions about specific preventive measures (for example, about wearing masks) are based on summarizing large complex datasets into small summarized numbers with limited conclusions. Therefore, it may cover significant details about health behavior and perhaps lead researchers to inaccurate conclusions or compromise the research validity [49].

## 5. NLP for Surveillance and Outbreak Prediction Systems

Social media-based infoveillance (i.e., information surveillance similar to syndromic surveillance that analyzes online data to detect disease outbreaks earlier than traditional surveillance [58,59]) has shown great potential in health applications [60,61]. For example, Chew and Eysenbach [62] analyzed tweets to monitor the use of the terms “H1N1” versus “swine flu” over time to study whether Twitter data can provide insights in predicting a pandemic, and thus, be used as an early tracking tool. Similarly, an influenza surveillance system has been proposed based on data extracted from Twitter [63]. Recent studies showed the potential of using social media data for developing pandemic prediction models based on early self-reported symptoms by users [64,65]. Moreover, an earlier systematic review [66] concluded that social media data are a valuable resource to develop syndromic surveillance systems that can detect infectious disease outbreaks by studying users’ spatiotemporal dynamics of self-reported symptoms. Such surveillance can work best when integrated with traditional systems. Likewise, early warnings of COVID-19 outbreaks across Europe have been detected from social media [67], showing that the number of reports of pneumonia was above usual in several European countries. Many of these social media posts came from geographical locations that later became COVID-19 hot spots (with high numbers of cases). All these studies suggest that social media-based infoveillance methods that utilize NLP can be critical for detecting early warning signals by analyzing online discussions.

## 6. NLP for Fighting Misinformation

The term *infodemic* refers to the massive information epidemic that occurs during a pandemic [5,68]. In recent years, the magnitude of this phenomenon has become large-scale with the continued popularity and adoption of social media platforms, which provides the means to spread information to an unprecedented number of users. Such information spreads without real-time verification and there is no effective mechanism for controlling it. Therefore, the combination of rapid emergent events such as COVID-19 and millions of connected users on social media can result in significantly amplified rumors and questionable information. The pressing need to develop AI that can fight the spread of misinformation has substantially increased with the COVID-19 pandemic, and this topic has received significant attention from governments and public health organizations [69,70]. One of the notable potential applications of NLP methods is to automatically detect misinformation that is spread over social media, such as fake news, rumors, hoaxes, and conspiracy theories.

Several NLP pipelines have been proposed to reduce the effect of the spread of misinformation [71,72,73]. For instance, a system to fight misinformation was developed using a dataset called ReCOVery (which contains multimodal information on COVID-19 news articles) and a similarity-aware multimodal fake news detection system (SAFE) [71]. The best classification results were used to distinguish between reliable and unreliable news [72]. Similarly, an NLP pipeline was annotated and developed using a support vector machine classifier to detect fake news related to COVID-19 [73]. Another study introduced a dataset that contained approximately 4800 tweets annotated by experts as informative, misinformative, or irrelevant. The authors applied off-the-shelf NLP models to the created dataset and concluded that the performances were not as good as required, suggesting the need for additional research and development. However, the current created dataset is small, with 165 informative and 465 misinformative tweets. Additional annotated data or augmentation may significantly improve the results. Other datasets include one on misinformation related to COVID-19, called CoAID, which contains 4251 news items, 296,000 related user engagements, and 926 posts from social networks with their ground truth labels [74], and another with 61,286 tweets related to the health-risk assessment of COVID-19. The latter mainly focuses on the severity of each misinformation story (the risk that a message is actually believed by the readers) [75].

NLP methods may also be combined with other computational approaches, such as complex networks, to discover hidden patterns and differences between the communities involved in spreading misinformation and promoting accurate information during the pandemic. For example, the literature has reported that communities that contribute to circulating misinformation are denser and more organized than those circulating useful information, with a possibility of a high volume of misinformation being part of disinformation campaigns [76]. COVID-19 users who spread useful information also tend to share more narratives than those who spread misinformation. NLP pipelines have also been developed to detect fake news related to COVID-19 through two stages [77]. In this approach, the first NLP stage uses a fact-checking method that retrieves the most relevant facts about particular COVID-19 claims. The second stage verifies the degree of truth in those claims. For these models, large pre-trained language models such as BERT and ALBERT were trained for the above proposal, and promising results were achieved in retrieving and classifying fake news in an extremely specific domain of COVID-19.

The studies demonstrated that NLP models can accurately identify the misinformation content primarily driven by known rumors, stigma, and conspiracy theories. However, most NLP models are built on supervised ML approaches that need to define in advance what needs to be detected via annotated samples. Therefore, such NLP models will likely miss most of the novel and unique misinformation content. Another challenge in building NLP-based solutions to mitigate the spread of misinformation is updating the NLP models with new and novel misinformation content to identify them promptly before they go viral. A potential future direction in misinformation identification is constructing lifelong learning strategies for NLP models to learn from crowdsourced judgment annotation [78], and ensuring that the learning process is timely.

## 7. NLP for COVID-19 Question-Answering Systems

Since the beginning of the COVID-19 outbreak, academics and researchers have focused on investigating COVID-19 and publishing relevant discoveries. The resulting large amount of published knowledge causes information overload [79], making it challenging for clinicians, medical professionals, and general readers to stay up to date with actionable insights. Real-time answers to important questions such as how the virus is transmitted, effective strategies for prevention, and risk factors for infection are essential and updated in almost real-time. Moreover, significant evidence needs to be summarized accordingly and conveyed to the public in a timely manner. Therefore, real-time question-answering (QA) systems based on the scientific literature can effectively disseminate information during an urgent time such as the COVID-19 pandemic.

To provide a large number of researchers and the public access to scientific findings on COVID-19, the World Health Organization (WHO), European Commission, and scientific research publishers have made relevant publications open access [5,80].

For COVID-19 QA and automatic text summarization (ATS), the common datasets that are available to researchers are as follows:

I. COVID-19 Open Research Dataset (CORD-19) [80]: A recent initiative established by the Allen Institute for AI, which contains all COVID-19-related publications. The CORD-19 dataset is updated daily to include the latest relevant published papers from various databases (such as arXiv, bioRxiv, and medRxiv, Medline, and PubMed Central) [80,81]. CORD-19 has more than 160,000 articles, of which more than 70,000 are full text [5]. The motive behind releasing this dataset is “to mobilize researchers to apply for recent advances in NLP to produce new insights in support of the fight against this infectious disease” [80].
II. COVID-QA dataset [82]: This dataset was created from scientific articles related to COVID-19 and annotated by volunteer biomedical experts. COVID-QA contains 2019 questions-and-answer pairs.
III. COVID-QA dataset by [83]: This dataset contains 124 question-and-article pairs annotated from the CORD-19 dataset.

Manual summarization is expensive and impractical. In practice, a manual summarization or search for an answer is impractical in the presence of massive amounts of textual data. ATS and QA systems hold a promising and practical solution to extract insights from such massive textual data. Researchers responding to the urgent call for building such solutions have developed ATS and QA systems. One of the first QA systems built using the CORD-19 corpus is CovidQA [83], for which the authors evaluated transformer models and unsupervised (zero-shot) approaches. The transformer models were proven effective for domain-specific supervised learning settings but had limited usefulness for out-of-domain contexts [83]. The analysis of several transformer models showed that T5 for ranking [84,85] accomplished the highest effectiveness in recognizing sentences from documents that contained answers.

Another research article [27] discussed the development of a real-time neural QA and query-focused multi-document summarization system called CAiRE-COVID. The system initially starts with the most relevant documents related to the input user query from the CORD-19 dataset and highlights the text spans containing the potential answer. The main NLP models used for building the CaiRE-COVID system architecture are as follows: a combination of two QA models, HLTC-MRQA [86] and BioBERT [87], are employed to construct the neural QA model; BART [88] for abstractive summarization; and ALBERT [89] in extractive summarization block. BERT is also used with topic modeling through latent Dirichlet allocation (LDA) to extract articles related to domains and retrieve answers to COVID-19 questions [90]. A real-time QA system that uses both biomedical text mining and QA methods to answer COVID-19-related questions was developed and called COVIDASK [91]. The primary NLP model in this architecture is BioBERT [87]. In other related research efforts, QA examples were synthetically generated to optimize the system performance on closed domains [92]. Neural information retrieval and machine reading comprehension methods were combined. The proposed approach showed significant increases in the performance of end-to-end QA on the CORD-19 collection compared with a state-of-the-art open-domain QA baseline.

Current QA systems, however, need further improvement to be used effectively during a pandemic. One of the primary challenges, mainly in the medical domain, is how to design QA systems that can respond with “*I do not know*” when a question is unanswerable or when an answer is uncertain. Moreover, while constructing QA systems, a follow-up question strategy to ask additional questions and information before providing the final answer, mainly when dealing with the complex question about COVID-19, is needed to avoid the ambiguity that may result in an inaccurate response [93]. QA systems should also include knowledge (e.g., common sense) beyond context-specific text and questions to which more accurate answers can be provided.

## 8. NLP for Knowledge Transfer

In response to the COVID-19 pandemic, universities and research centers conducted studies to understand the nature of the new virus, its transmission, risk factors, preventive steps, and measures to increase community awareness and prepare official guidelines. However, most of the published scientific reports and articles are in English, and translation of the scientific findings into several other languages is necessary to reach a larger population worldwide. NLP can play an important role to translate these findings and guidelines. For instance, NLP models were trained to offer multilingual translation support for general and biomedical domains [94]. A separate study constructed a multilingual dataset and then developed a model for cross-lingual intent detection to improve COVID-19 chatbots across the English, Spanish, French, and Spanglish languages [95]. Multilingual models have also been developed to understand people’s sentiments about COVID-19 across various languages and countries [96].

Table 1 summarizes the important studies discussed in the previous sections and compares the NLP methods used in various applications related to COVID-19. The comparison confirmed that the pre-trained NLP models, such as BERT, ALBERT, Sentence-BERT, and Bio-BERT, are commonly used NLP models for building NLP pipelines for COVID-19.

Table 1 shows a comparison of the NLP methods on various applications related to COVID-19.

## 9. Opportunities and Challenges for NLP Applications during the COVID-19 Pandemic

The potential of NLP-based technologies is coupled with challenges associated with their development and application. In this section, we present the challenges in using NLP approaches to help mitigate the impacts of pandemics and improve pandemic preparedness. The challenges discussed are related to the nature of pandemics, the design of clinically applicable NLP models, sampling bias, data analysis, characteristics of health misinformation, synergic implementation, and deployment.

### 9.1. The Nature of a Pandemic

Pandemics are large-scale infectious disease outbreaks that can cause a critical upsurge in infection spread and mortality over a wide-ranging geographical region, leading to significant economic, social, and political disruptions [104,105]. The probability of pandemic occurrence has increased over the past century because of globalization, urbanization, changes in land use, and extensive exploitation of the natural environment [104,105]. Thus, improving our capability to respond to pandemics remains a challenge. COVID-19 is transmitted quite easily, with the average infected person spreading the disease to two or three others [106], and some recently emerging variants such as Delta and Omicron are even more infectious [107]. The rapid spread of COVID-19 necessitates the need for fast responses. However, developing NLP models that can efficiently support healthcare response systems still faces many obstacles. Most current successful NLP models are trained on manually annotated data, which is time-consuming to create. Moreover, many annotated datasets, particularly those involving EHRs, are not publicly shared and are confined within the specific institution that is conducting the research. The lack of mechanisms for widespread data sharing presents challenges related to the generalization of implemented systems. Many systems that are developed remain effective only within the creating organization and typically underperform when applied to other healthcare settings. Creating frameworks that can enhance the data-annotation processes and enable widespread knowledge sharing can address such challenges and help develop NLP models that promptly meet the needs of people during the pandemic.

### 9.2. Characteristics of Health Misinformation

One potential application of NLP models is combatting the spread of health misinformation during the pandemic. However, misinformation is written in a manner that presents difficulties for the public to distinguish it from correct information [108]. Moreover, misinformation occurs as a distributed incident and usually spreads faster than the correct information [109] with dynamic modification to avoid automated detection [108,109]. This issue can increase the difficulty of designing an NLP model to detect such dynamic spread.

The above challenges can be mitigated by designing NLP models that can speedily detect changes in public priorities, therefore, providing the necessary accurate information in a timely manner. Patterns and knowledge derived from social media can be used to guide targeted interventions [110]. Timely identification of the information discussed in subsets of populations can lead to more specific data campaigns and earlier public awareness of spreading misinformation [110].

### 9.3. Designing Clinically Applicable NLP Models

NLP models can be designed to extract actionable information by combining AI and clinical research [111]. On the one hand, the design of such systems must be clinically useful, and on the other hand, they must be implementable by NLP researchers who are typically not medical domain experts. An advantage of using NLP in healthcare is automation; clinicians cannot process data as rapidly as machines. Nevertheless, automated systems are trained and evaluated on selected databases that only contain information that may be specific to a targeted cohort or geolocation. If the databases do not represent the complete set of potential circumstances, then the automated systems can make incorrect decisions in cases that have never been examined [112].

The risk of inaccurate models is remarkably higher than that of a single doctor–patient interaction, yet the advantages of reducing cost, human errors, and inefficiencies in current healthcare systems are substantial [23]. One potential mechanism by which risks of AI or NLP-related errors can be mitigated is through the development of interpretable models. In this case, interpretability needs to focus on the medical practitioners who should be able to view the reasoning behind system decisions and decide if the system’s recommendations/decisions should be used. For critical clinical decisions, NLP researchers need to construct accurate but interpretable models that can identify the patterns that clinicians find interpretable, yet they should also be robust to make accurate decisions [112].

### 9.4. Synergic Implementation and Deployment

NLP systems can be most beneficial when incorporated into healthcare and public health systems. Digital health data (EHRs, scientific research findings, health information in social media) can be combined and processed in the NLP systems that benefit from each data source to provide recommendations on the individual and population levels. In the future, healthcare systems that can link clinical notes across different institutions must be developed to provide clinicians with tools to automate tasks and extract useful information. The NLP of scientific research can provide clinicians with timely and accurate updates, and social media can be used for outreach, crowdsourcing information, surveillance, and fighting misinformation. Ideally, such an NLP system can work on various data sources but still serve the ultimate goal of decreasing the consequences of outbreaks in society.

### 9.5. Sampling Bias on Social Media

Social media is a crucial data source to understand the impact of COVID-19 on subsets of populations. However, conducting social media-based studies, such as on mental health, can introduce sampling bias. Social media users are more likely to be younger and technologically savvy, resulting in biased samples. However, the wealth and diversity of accessible content make social media attractive as a data source [100]. Additionally, according to PEW research [113], the adoption of social media is growing among older populations, which means that in the future, it will be better representative of populations.

### 9.6. Data Analysis Challenge

NLP methods for studying health behavior, conducting pandemic surveillance, and monitoring mental health status at large scales can provide more comprehensive findings and insights than traditional approaches. The main objective is to translate the textual content into insightful statistical numbers (e.g., numbers of positive/negative posts, the intensity of positivity/negativity or emotion in a post, or a number of self-reported COVID-19 cases). However, researchers tend to aggregate statistical numbers to make them more manageable and perform overall descriptive analysis. How this aggregation of numerical findings is accomplished can compromise the final findings and may provide incorrect interpretations [49]. For example, when aggregating the number of positive or negative sentiments to study the sentiment changes during the COVID-19 phase, the number of positive or negative posts may give weight to active users’ sentiments in the final inference, which in turn may lead to a biased conclusion toward these sentiments, rather than a conclusion derived from the overall population.

## 10. Conclusions

This review presented a set of important uses of NLP approaches that have the potential to improve pandemic preparedness and response during a pandemic. NLP has great potential at a time when huge amounts of text-based data are constantly created from sources such as healthcare systems, the scientific literature, and health social media. In this review, we emphasized the potential utility of NLP for addressing many pandemic-specific problems, including the swift execution of pandemic responses at scale and low cost. We described the lessons learned for each NLP application, including the capabilities and limitations of existing NLP methods and how they may be utilized to improve health and social care. We provided summary tables for the highlighted studies discussed in the previous sections and presented comparisons of NLP methods on various applications related to COVID-19. The comparison tables specifically show that pre-trained NLP models, such as BERT, ALBERT, Sentence-BERT, and Bio BERT, are perhaps the most commonly used elements by NLP pipelines. We highlighted the key challenges associated with the use of NLP pipelines as parts of diverse pandemic response systems; these challenges include the nature of pandemics, designing clinically applicable NLP models, sampling bias, data analysis challenges, characteristics of health misinformation, synergic implementation, and deployment-related issues. The identified challenges and related opportunities can serve as potential future research directions.

## Figures and Tables

**Table 1:** Comparison of studies that apply NLP to various applications related to COVID-19.

Study Reference	Application	Employed NLP Model	Data Source
[30]	EHRs	- BERT - Specific phenotypes associated with COVID-19 using the list of 60 regular expressions (NLP RegExp) - All signs, symptoms, and comorbidities were extracted with the quickUMLS algorithm [97] (NLP UMLS).	A multi-center study involving data from 39 hospitals
[98]	EHRs	Keyword-extraction NLP that uses an unsupervised ML approach (clustering)	450,114 patient CT comprehensive reports gathered from 1 January to October 2020
[99]	EHRs	Word frequency for text analytics and CNN trained using Word2vector as a classification model	Data are collected through telehealth visits, including 6813 patients, of whom 498 tested positive and 6315 tested negative
[32]	EHRs	NLP model (medical named entity recognition)	Audio or video recordings of clinic visits
[38]	EHRs	Multi-class logistic regression model trained n-gram features	The study cohort includes 1737 COVID-19 adult patients discharged from two hospitals in Boston, Massachusetts, between 10 March and 30 June 2022
[39]	EHRs	NLP rule-based pipeline	Data from VA Corporate Data Warehouse (CDW) include clinical data in 2020 between 1 January and 15 June
[33]	EHRs	Random-forest trained on N-grams	32,555 radiology reports from brain CTs and MRIs from a comprehensive stroke center
[34]	EHRs	NLP rule-based pipeline	6250 patients (5664 negative and 586 positives; 46,138 non-severe and 125 severe)
[36]	EHRs	BERT and Bi-LSTM with attention	Annotated 1472 clinical notes distinguishing COVID-19 diagnoses, testing, and symptoms
[35]	EHRs	NLP rule-based pipeline	NLP is validated on several datasets; the main one is related to COVID-19 and contains 50 posts (1162 sentences) of related dialogues
[44]	Mental health	Supervised text classification used stochastic gradient descent linear classifier with L1 penalty TF-IDF grams with principal component analysis with k-NN used for unsupervised clustering. LDA is used in topic modeling.	Social media: Reddit Mental Health Dataset including posts from 826,961 unique users
[45]	Mental health	BERT (ft)	Social media: 1000 English tweets for training the model and 1 million tweets included in the analysis
[46]	Mental health	Sentiment analytic systems called CrystalFeel	Social media: Over 20 million COVID-19 tweets between 28 January and April 2020
[47]	Mental health	Key phrase extraction and sentiment score using lexicon-based technique	Social media: 47 million COVID-19- related comments extracted from Twitter, Facebook, and YouTube
[100]	Mental health	Bi-directional LSTM and a self-attention layer	Social media: The diagnosed group has approximately 900,000 tweets from several countries. The control group has approximately 14 million tweets from several countries
[48]	Mental health	Sentence-BERT (SBERT)	9090 English free-form texts from 1451 students between 1 February and 30 April 2020
[52]	Health behaviors	BERT	1.1 million COVID-19-related tweets from 181 counties in the US
[54]	Health behaviors	- t-Distributed Stochastic Neighbor Embedding - DistilBART - VADER for sentiment analysis - Google’s Universal Sentence Encoder	189,958,459 English COVID-19-related tweets COVID-19 between 17 March to 27 July 2020
[55]	Health behaviors	SVM, XGBoost, and LSTM	771,268 tweets from the US between January and October 2020
[56]	Health behaviors	LDA for topic modeling andaspect-based sentiment analysis	English COVID-19 tweets are 25,595 for Canada and 293,929 for the US
[57]	Health behaviors	BERT	2,349,659 tweets related to COVID-19 vaccination 1 month after the first vaccine announcement
[52]	Health behaviors	BERT	1.1 million COVID-19-related tweets from 181 counties in the US
[71]	Misinformation detection	Uses SAFE systems developed in [53]	2029 news articles on COVID-19 (between January and May 2020) and 140,820 tweets that disclose how these news articles have circulated on Twitter
[76]	Misinformation detection	NLP and network analysis method	4573 annotated tweets comprising 3629 users
[73]	Misinformation detection	SVM	10,700 social media posts and articles of real and fake news on COVID-19
[101]	Misinformation detection	Sentence-BERT and BERTScore	4800 expert-annotated social media posts
[77]	Misinformation detection	BERT and ALBERT	5500 claims and explanation pairs
[90]	COVID QA systems	BERT and LDA	COVID-19 scientific publications: CORD-19 dataset
[83]	COVID QA systems	T5	COVID-19 scientific publications: CORD-19 dataset
[102]	COVID QA systems	- An ensemble of two QA models (HLTC-MRQA and BioBERT) for the QA model - BART [88] for abstractive summarization - ALBERT [89] in extractive summarization block	COVID-19 scientific publications: CORD-19 dataset
[91]	COVID QA systems	BioBERT	COVID-19 scientific publications: CORD-19 dataset, with additional 111 QA pairs annotated for test
[92]	COVID QA systems	Synthetically generated QA examples to optimize the QA system performance on closed domains. The machine reading comprehension employs the Roberta model.	COVID-19 scientific publications: CORD-19 dataset
[95]	Knowledge transfer	XLM-R Large	Dataset, M-CID, containing 5271 utterances across English, Spanish, French, and Spanglish
[96]	Knowledge transfer	Multilingual Universal Sentence Encoder [103]	4,683,226 geo-referenced tweets in 60 languages located in Europe
[94]	Knowledge transfer	Variant transformers big architecture	The model is trained on more than 350 million sentences in French, Spanish, German, Italian, and Korean (into English)

## Data Availability

Not applicable.

## References

[1] Legido-Quigley H., Asgari N., Teo Y.Y., Leung G.M., Oshitani H., Fukuda K., Cook A.R., Hsu L.Y., Shibuya K., Heymann D. (2020). Are high-performing health systems resilient against the COVID-19 epidemic?. Lancet.

[2] El Bcheraoui C., Weishaar H., Pozo-Martin F., Hanefeld J. (2020). Assessing COVID-19 through the lens of health systems’ preparedness: Time for a change. Glob. Health.

[3] Budd J., Miller B.S., Manning E.M., Lampos V., Zhuang M., Edelstein M., Rees G., Emery V.C., Stevens M.M., Keegan N. (2020). Digital technologies in the public-health response to COVID-19. Nat. Med..

[4] Venkatakrishnan A., Pawlowski C., Zemmour D., Hughes T., Anand A., Berner G., Kayal N., Puranik A., Conrad I., Bade S. (2021). Mapping each pre-existing condition’s association to short-term and long-term COVID-19 complications. Npj Digit. Med..

[5] Zarocostas J. (2020). How to fight an infodemic. Lancet.

[6] Yan R., Liao W., Cui J., Zhang H., Hu Y., Zhao D. Multilingual COVID-QA: Learning towards global information sharing via web question answering in multiple languages. Proceedings of the Web Conference 2021.

[7] Liu H., Liu W., Yoganathan V., Osburg V.-S. (2021). COVID-19 information overload and generation Z’s social media discontinuance intention during the pandemic lockdown. Technol. Forecast. Soc. Chang..

[8] Poonia S.K., Rajasekaran K. (2020). Information overload: A method to share updates among frontline staff during the COVID-19 pandemic. Otolaryngol. -Head Neck Surg..

[9] Grabar N., Grouin C. (2021). Year 2020 (with COVID): Observation of Scientific Literature on Clinical Natural Language Processing. Yearb. Med. Inform..

[10] Guo Y., Zhang Y., Lyu T., Prosperi M., Wang F., Xu H., Bian J. (2021). The application of artificial intelligence and data integration in COVID-19 studies: A scoping review. J. Am. Med. Inform. Assoc..

[11] Chen Q., Leaman R., Allot A., Luo L., Wei C.-H., Yan S., Lu Z. (2021). Artificial intelligence in action: Addressing the COVID-19 pandemic with natural language processing. Annu. Rev. Biomed. Data Sci..

[12] Hallak J.A., Scanzera A., Azar D.T., Chan R.P. (2020). Artificial intelligence in ophthalmology during COVID-19 and in the post COVID-19 era. Curr. Opin. Ophthalmol..

[13] Chatterjee A., Nardi C., Oberije C., Lambin P. (2021). Knowledge Graphs for COVID-19: An Exploratory Review of the Current Landscape. J. Pers. Med..

[14] Abd-Alrazaq A., Alajlani M., Alhuwail D., Schneider J., Al-Kuwari S., Shah Z., Hamdi M., Househ M. (2020). Artificial intelligence in the fight against COVID-19: Scoping review. J. Med. Internet Res..

[15] Tsao S.-F., Chen H., Tisseverasinghe T., Yang Y., Li L., Butt Z.A. (2021). What social media told us in the time of COVID-19: A scoping review. Lancet Digit. Health.

[16] Chen J., Wang Y. (2021). Social Media Use for Health Purposes: Systematic Review. J. Med. Internet Res..

[17] Shorten C., Khoshgoftaar T.M., Furht B. (2021). Deep Learning applications for COVID-19. J. Big Data.

[18] Lalmuanawma S., Hussain J., Chhakchhuak L. (2020). Applications of machine learning and artificial intelligence for COVID-19 (SARS-CoV-2) pandemic: A review. Chaos Solitons Fractals.

[19] Islam M.M., Karray F., Alhajj R., Zeng J. (2021). A review on deep learning techniques for the diagnosis of novel coronavirus (COVID-19). IEEE Access.

[20] De Felice F., Polimeni A. (2020). Coronavirus disease (COVID-19): A machine learning bibliometric analysis. In Vivo.

[21] Alzubaidi M., Zubaydi H.D., Bin-Salem A.A., Abd-Alrazaq A.A., Ahmed A., Househ M. (2021). Role of deep learning in early detection of COVID-19: Scoping review. Comput. Methods Programs Biomed. Update.

[22] Hall K., Chang V., Jayne C. (2022). A review on Natural Language Processing Models for COVID-19 research. Healthc. Anal..

[23] Topol E.J. (2019). High-performance medicine: The convergence of human and artificial intelligence. Nat. Med..

[24] Esteva A., Chou K., Yeung S., Naik N., Madani A., Mottaghi A., Liu Y., Topol E., Dean J., Socher R. (2021). Deep learning-enabled medical computer vision. Npj Digit. Med..

[25] Wang F., Casalino L.P., Khullar D. (2019). Deep learning in medicine—Promise, progress, and challenges. JAMA Intern. Med..

[26] Locke S., Bashall A., Al-Adely S., Moore J., Wilson A., Kitchen G.B. (2021). Natural language processing in medicine: A review. Trends Anaesth. Crit. Care.

[27] Ching T., Himmelstein D.S., Beaulieu-Jones B.K., Kalinin A.A., Do B.T., Way G.P., Ferrero E., Agapow P.-M., Zietz M., Hoffman M.M. (2018). Opportunities and obstacles for deep learning in biology and medicine. J. R. Soc. Interface.

[28] Van der Laak J., Litjens G., Ciompi F. (2021). Deep learning in histopathology: The path to the clinic. Nat. Med..

[29] Ohno-Machado L. (2011). Realizing the full potential of electronic health records: The role of natural language processing. J. Am. Med. Inform. Assoc..

[30] Neuraz A., Lerner I., Digan W., Paris N., Tsopra R., Rogier A., Baudoin D., Cohen K.B., Burgun A., Garcelon N. (2020). Natural language processing for rapid response to emergent diseases: Case study of calcium channel blockers and hypertension in the COVID-19 pandemic. J. Med. Internet Res..

[31] Elkin P.L., Froehling D.A., Wahner-Roedler D.L., Brown S.H., Bailey K.R. (2012). Comparison of natural language processing biosurveillance methods for identifying influenza from encounter notes. Ann. Intern. Med..

[32] Barr P.J., Ryan J., Jacobson N.C. (2021). Precision Assessment of COVID-19 Phenotypes Using Large-Scale Clinic Visit Audio Recordings: Harnessing the Power of Patient Voice. J. Med. Internet Res..

[33] Li M., Lang M., Deng F., Chang K., Buch K., Rincon S., Mehan W., Leslie-Mazwi T., Kalpathy-Cramer J. (2021). Analysis of stroke detection during the COVID-19 pandemic using natural language processing of radiology reports. Am. J. Neuroradiol..

[34] Schoening V., Liakoni E., Drewe J., Hammann F. (2021). Automatic identification of risk factors for SARS-CoV-2 positivity and severe clinical outcomes of COVID-19 using Data Mining and Natural Language Processing. medRxiv.

[35] Wang J., Abu-el-Rub N., Gray J., Pham H.A., Zhou Y., Manion F.J., Liu M., Song X., Xu H., Rouhizadeh M. (2021). COVID-19 SignSym: A fast adaptation of a general clinical NLP tool to identify and normalize COVID-19 signs and symptoms to OMOP common data model. J. Am. Med. Inform. Assoc..

[36] Lybarger K., Ostendorf M., Thompson M., Yetisgen M. (2021). Extracting COVID-19 diagnoses and symptoms from clinical text: A new annotated corpus and neural event extraction framework. J. Biomed. Inform..

[37] Izquierdo J.L., Ancochea J., Soriano J.B., Group S.C.-R. (2020). Clinical characteristics and prognostic factors for intensive care unit admission of patients With COVID-19: Retrospective study using machine learning and natural language processing. J. Med. Internet Res..

[38] Fernandes M., Sun H., Jain A., Alabsi H.S., Brenner L.N., Ye E., Ge W., Collens S.I., Leone M.J., Das S. (2021). Classification of the Disposition of Patients Hospitalized with COVID-19: Reading Discharge Summaries Using Natural Language Processing. JMIR Med. Inform..

[39] Chapman A.B., Peterson K.S., Turano A., Box T.L., Wallace K.S., Jones M. (2020). A Natural Language Processing System for National COVID-19 Surveillance in the US Department of Veterans Affairs. Openreview.

[40] Pfefferbaum B., North C.S. (2020). Mental health and the COVID-19 pandemic. N. Engl. J. Med..

[41] Xiong J., Lipsitz O., Nasri F., Lui L.M., Gill H., Phan L., Chen-Li D., Iacobucci M., Ho R., Majeed A. (2020). Impact of COVID-19 pandemic on mental health in the general population: A systematic review. J. Affect. Disord..

[42] Calvo R.A., Milne D.N., Hussain M.S., Christensen H. (2017). Natural language processing in mental health applications using non-clinical texts. Nat. Lang. Eng..

[43] Abd Rahman R., Omar K., Noah S.A.M., Danuri M.S.N.M., Al-Garadi M.A. (2020). Application of machine learning methods in mental health detection: A systematic review. IEEE Access.

[44] Low D.M., Rumker L., Talkar T., Torous J., Cecchi G., Ghosh S.S. (2020). Natural Language Processing Reveals Vulnerable Mental Health Support Groups and Heightened Health Anxiety on Reddit During COVID-19: Observational Study. J. Med. Internet Res..

[45] Li I., Li Y., Li T., Alvarez-Napagao S., Garcia-Gasulla D., Suzumura T., Bramer M., Ellis R. What Are We Depressed About When We Talk About COVID-19: Mental Health Analysis on Tweets Using Natural Language Processing. Artificial Intelligence XXXVII, Proceedings of the 40th SGAI International Conference on Artificial Intelligence, AI 2020, Cambridge, UK, 15–17 December 2020.

[46] Lwin M.O., Lu J., Sheldenkar A., Schulz P.J., Shin W., Gupta R., Yang Y. (2020). Global sentiments surrounding the COVID-19 pandemic on Twitter: Analysis of Twitter trends. JMIR Public Health Surveill..

[47] Oyebode O., Ndulue C., Adib A., Mulchandani D., Suruliraj B., Orji F.A., Chambers C., Meier S., Orji R. (2021). Health, Psychosocial, and Social issues emanating from COVID-19 pandemic based on Social Media Comments using Text Mining and Thematic Analysis. JMIR Med. Inform..

[48] Sharma R., Pagadala S.D., Bharti P., Chellappan S., Schmidt T., Goyal R. (2020). Assessing COVID-19 Impacts on College Students via Automated Processing of Free-form Text. arXiv.

[49] Olteanu A., Castillo C., Diaz F., Kıcıman E. (2019). Social data: Biases, methodological pitfalls, and ethical boundaries. Front. Big Data.

[50] Howison J., Wiggins A., Crowston K. (2011). Validity issues in the use of social network analysis with digital trace data. J. Assoc. Inf. Syst..

[51] Chancellor S., De Choudhury M. (2020). Methods in predictive techniques for mental health status on social media: A critical review. Npj Digit. Med..

[52] Verspoor K., Cohen K.B., Conway M., De Bruijn B., Dredze M., Mihalcea R., Wallace B.C. Proceedings of the 1st Workshop on NLP for COVID-19 (Part 2) at EMNLP 2020.

[53] Kwon J., Grady C., Feliciano J.T., Fodeh S.J. (2020). Defining facets of social distancing during the COVID-19 pandemic: Twitter analysis. J. Biomed. Inform..

[54] Sanders A.C., White R.C., Severson L.S., Ma R., McQueen R., Paulo H.C.A., Zhang Y., Erickson J.S., Bennett K.P. (2021). Unmasking the conversation on masks: Natural language processing for topical sentiment analysis of COVID-19 Twitter discourse. AMIA Summits Transl. Sci. Proc..

[55] He L., He C., Reynolds T.L., Bai Q., Huang Y., Li C., Zheng K., Chen Y. (2021). Why do people oppose mask wearing? A comprehensive analysis of US tweets during the COVID-19 pandemic. J. Am. Med. Inform. Assoc..

[56] Jang H., Rempel E., Roth D., Carenini G., Janjua N.Z. (2021). Tracking COVID-19 Discourse on Twitter in North America: Infodemiology Study Using Topic Modeling and Aspect-Based Sentiment Analysis. J. Med. Internet Res..

[57] Cotfas L.-A., Delcea C., Roxin I., Ioanăş C., Gherai D.S., Tajariol F. (2021). The Longest Month: Analyzing COVID-19 Vaccination Opinions Dynamics From Tweets in the Month Following the First Vaccine Announcement. IEEE Access.

[58] Eysenbach G. Infodemiology: Tracking Flu-Related Searches on the Web for Syndromic Surveillance. AMIA Annual Symposium Proceedings.

[59] Velardi P., Stilo G., Tozzi A.E., Gesualdo F. (2014). Twitter mining for fine-grained syndromic surveillance. Artif. Intell. Med..

[60] Eysenbach G. (2009). Infodemiology and infoveillance: Framework for an emerging set of public health informatics methods to analyze search, communication and publication behavior on the Internet. J. Med. Internet Res..

[61] Brownstein J.S., Freifeld C.C., Madoff L.C. (2009). Digital disease detection—Harnessing the Web for public health surveillance. N. Engl. J. Med..

[62] Chew C., Eysenbach G. (2010). Pandemics in the age of Twitter: Content analysis of Tweets during the 2009 H1N1 outbreak. PLoS ONE.

[63] Broniatowski D.A., Paul M.J., Dredze M. (2013). National and local influenza surveillance through Twitter: An analysis of the 2012-2013 influenza epidemic. PLoS ONE.

[64] Lampos V., Cristianini N. Tracking the Flu Pandemic by Monitoring the Social Web. Proceedings of the 2010 2nd International Workshop on Cognitive Information Processing.

[65] Neumann G., Kawaoka Y. (2019). Predicting the next influenza pandemics. J. Infect. Dis..

[66] Al-Garadi M.A., Khan M.S., Varathan K.D., Mujtaba G., Al-Kabsi A.M. (2016). Using online social networks to track a pandemic: A systematic review. J. Biomed. Inform..

[67] Lopreite M., Panzarasa P., Puliga M., Riccaboni M. (2021). Early warnings of COVID-19 outbreaks across Europe from social media. Sci. Rep..

[68] Cinelli M., Quattrociocchi W., Galeazzi A., Valensise C.M., Brugnoli E., Schmidt A.L., Zola P., Zollo F., Scala A. (2020). The COVID-19 social media infodemic. Sci. Rep..

[69] WHO (2020). Novel Coronavirus (2019-nCoV) Situation Report—13.

[70] Tasnim S., Hossain M.M., Mazumder H. (2020). Impact of rumors and misinformation on COVID-19 in social media. J. Prev. Med. Public Health.

[71] Zhou X., Wu J., Zafarani R. (2020). (SAFE): Similarity-Aware Multi-modal Fake News Detection. Pacific-Asia Conference on Knowledge Discovery and Data Mining.

[72] Zhou X., Mulay A., Ferrara E., Zafarani R. Recovery: A Multimodal Repository for COVID-19 News Credibility Research. Proceedings of the 29th ACM International Conference on Information & Knowledge Management.

[73] Patwa P., Sharma S., PYKL S., Guptha V., Kumari G., Akhtar M.S., Ekbal A., Das A., Chakraborty T. (2020). Fighting an infodemic: COVID-19 fake news dataset. arXiv.

[74] Cui L., Lee D. (2020). Coaid: COVID-19 healthcare misinformation dataset. arXiv.

[75] Dharawat A., Lourentzou I., Morales A., Zhai C. (2020). Drink bleach or do what now? Covid-HeRA: A dataset for risk-informed health decision making in the presence of COVID19 misinformation. arXiv.

[76] Memon S.A., Carley K.M. (2020). Characterizing COVID-19 misinformation communities using a novel twitter dataset. arXiv.

[77] Vijjali R., Potluri P., Kumar S., Teki S. (2020). Two stage transformer model for COVID-19 fake news detection and fact checking. arXiv.

[78] Pennycook G., Rand D.G. (2019). Fighting misinformation on social media using crowdsourced judgments of news source quality. Proc. Natl. Acad. Sci. USA.

[79] Rathore F.A., Farooq F. (2020). Information overload and infodemic in the COVID-19 pandemic. J. Pak. Med. Assoc..

[80] Colavizza G., Costas R., Traag V.A., Van Eck N.J., Van Leeuwen T., Waltman L. (2021). A scientometric overview of CORD-19. PLoS ONE.

[81] Wang L.L., Lo K., Chandrasekhar Y., Reas R., Yang J., Eide D., Funk K., Kinney R., Liu Z., Merrill W. (2020). Cord-19: The COVID-19 open research dataset. arXiv.

[82] Möller T., Reina A., Jayakumar R., Pietsch M. COVID-QA: A Question Answering Dataset for COVID-19. Proceedings of the ACL 2020 Workshop on Natural Language Processing for COVID-19 (NLP-COVID).

[83] Tang R., Nogueira R., Zhang E., Gupta N., Cam P., Cho K., Lin J. (2020). Rapidly bootstrapping a question answering dataset for COVID-19. arXiv.

[84] Raffel C., Shazeer N., Roberts A., Lee K., Narang S., Matena M., Zhou Y., Li W., Liu P.J. (2019). Exploring the limits of transfer learning with a unified text-to-text transformer. arXiv.

[85] Nogueira R., Jiang Z., Lin J. (2020). Document ranking with a pretrained sequence-to-sequence model. arXiv.

[86] Su D., Xu Y., Winata G.I., Xu P., Kim H., Liu Z., Fung P. Generalizing Question Answering System with Pre-Trained Language Model Fine-Tuning. Proceedings of the 2nd Workshop on Machine Reading for Question Answering.

[87] Lee J., Yoon W., Kim S., Kim D., Kim S., So C.H., Kang J. (2020). BioBERT: A pre-trained biomedical language representation model for biomedical text mining. Bioinformatics.

[88] Lewis M., Liu Y., Goyal N., Ghazvininejad M., Mohamed A., Levy O., Stoyanov V., Zettlemoyer L. (2019). Bart: Denoising sequence-to-sequence pre-training for natural language generation, translation, and comprehension. arXiv.

[89] Lan Z., Chen M., Goodman S., Gimpel K., Sharma P., Soricut R. (2019). Albert: A lite bert for self-supervised learning of language representations. arXiv.

[90] Venkataram H.S., Mattmann C.A., Penberthy S. TopiQAL: Topic-aware Question Answering using Scalable Domain-specific Supercomputers. Proceedings of 2020 IEEE/ACM Fourth Workshop on Deep Learning on Supercomputers (DLS).

[91] Lee J., Yi S.S., Jeong M., Sung M., Yoon W., Choi Y., Ko M., Kang J. (2020). Answering questions on COVID-19 in real-time. arXiv.

[92] Reddy R.G., Iyer B., Sultan M.A., Zhang R., Sil A., Castelli V., Florian R., Roukos S. (2020). End-to-End QA on COVID-19: Domain Adaptation with Synthetic Training. arXiv.

[93] Zhu F., Lei W., Wang C., Zheng J., Poria S., Chua T.-S. (2021). Retrieving and reading: A comprehensive survey on open-domain question answering. arXiv.

[94] Bérard A., Kim Z.M., Nikoulina V., Park E.L., Gallé M. (2020). A Multilingual Neural Machine Translation Model for Biomedical Data. arXiv.

[95] Arora A., Shrivastava A., Mohit M., Lecanda L.S.-M., Aly A. (2020). Cross-lingual Transfer Learning for Intent Detection of COVID-19 Utterances. Openreview.

[96] Kruspe A., Häberle M., Kuhn I., Zhu X.X. (2020). Cross-language sentiment analysis of European Twitter messages duringthe COVID-19 pandemic. arXiv.

[97] Okazaki N., Tsujii J.I. Simple and Efficient Algorithm for Approximate Dictionary Matching. Proceedings of the 23rd International Conference on Computational Linguistics (Coling 2010).

[98] Cury R.C., Megyeri I., Lindsey T., Macedo R., Batlle J., Kim S., Baker B., Harris R., Clark R.H. (2021). Natural language processing and machine learning for detection of respiratory illness by chest ct imaging and tracking of COVID-19 pandemic in the us. Radiol. Cardiothorac. Imaging.

[99] Obeid J.S., Davis M., Turner M., Meystre S.M., Heider P.M., O’Bryan E.C., Lenert L.A. (2020). An artificial intelligence approach to COVID-19 infection risk assessment in virtual visits: A case report. J. Am. Med. Inform. Assoc..

[100] Tabak T., Purver M. (2020). Temporal Mental Health Dynamics on Social Media. arXiv.

[101] Micallef N., He B., Kumar S., Ahamad M., Memon N. (2020). The Role of the Crowd in Countering Misinformation: A Case Study of the COVID-19 Infodemic. arXiv.

[102] Dan S., Xu Y., Yu T., Siddique F.B., Barezi E., Fung P. (2020). CAiRE-COVID: A question answering and query-focused multi-document summarization system for COVID-19 scholarly information management. arXiv.

[103] Yang Y., Cer D., Ahmad A., Guo M., Law J., Constant N., Abrego G.H., Yuan S., Tar C., Sung Y.-H. (2019). Multilingual universal sentence encoder for semantic retrieval. arXiv.

[104] Madhav N., Oppenheim B., Gallivan M., Mulembakani P., Rubin E., Wolfe N. (2017). Pandemics: Risks, Impacts, and Mitigation. Disease Control Priorities: Improving Health and Reducing Poverty.

[105] Jones K.E., Patel N.G., Levy M.A., Storeygard A., Balk D., Gittleman J.L., Daszak P. (2008). Global trends in emerging infectious diseases. Nature.

[106] Gates B. (2020). Responding to COVID-19—A once-in-a-century pandemic?. N. Engl. J. Med..

[107] CDC (2021). Delta Variant: What We Know About the Science. Cent. Dis. Control. Prev..

[108] de Oliveira N.R., Pisa P.S., Lopez M.A., de Medeiros D.S.V., Mattos D.M. (2021). Identifying Fake News on Social Networks Based on Natural Language Processing: Trends and Challenges. Information.

[109] Southwell B.G., Niederdeppe J., Cappella J.N., Gaysynsky A., Kelley D.E., Oh A., Peterson E.B., Chou W.-Y.S. (2019). Misinformation as a misunderstood challenge to public health. Am. J. Prev. Med..

[110] Stokes D.C., Andy A., Guntuku S.C., Ungar L.H., Merchant R.M. (2020). Public priorities and concerns regarding COVID-19 in an online discussion forum: Longitudinal topic modeling. J. Gen. Intern. Med..

[111] Wu J.T., Dernoncourt F., Gehrmann S., Tyler P.D., Moseley E.T., Carlson E.T., Grant D.W., Li Y., Welt J., Celi L.A. (2018). Behind the scenes: A medical natural language processing project. Int. J. Med. Inform..

[112] Rudin C. (2019). Stop explaining black box machine learning models for high stakes decisions and use interpretable models instead. Nat. Mach. Intell..

[113] Auxier B., Anderson M. (2021). Social Media Use in 2021. Pew Research Center. https://www.pewresearch.org/internet/wp-content/uploads/sites/9/2021/04/PI_2021.04.07_Social-Media-Use_FINAL.pdf.

